# Comparison of scientometric achievements at PhD and scientific output ten years later for 4,790 academic researchers

**DOI:** 10.1371/journal.pone.0271218

**Published:** 2022-07-27

**Authors:** Gyöngyi Munkácsy, Péter Herman, Balázs Győrffy

**Affiliations:** 1 Department of Bioinformatics and 2nd Dept. of Pediatrics, Semmelweis University, Budapest, Hungary; 2 TTK Lendület Cancer Biomarker Research Group, Institute of Enzymology, Budapest, Hungary; University of Naples L’Orientale, ITALY

## Abstract

**Introduction:**

PhD is the highest awarded degree offered by universities in different disciplines. Owners of a PhD can teach at universities, start independent research and receive a higher salary while further building a scientific career. We examined whether the publication output before the PhD degree has a correlation with subsequent research activities.

**Methods:**

We downloaded publication and citation data from the Hungarian Scientific Bibliography for Hungarian researchers who obtained PhD between the ages of 24 and 45. The researchers were grouped into eleven scientific sections. We examined the number of Q1 publications published in the previous 5 years, the H-index, the total number of citations for the last complete year, and the biological age of the researcher. Each parameter was computed for the year at which the PhD was obtained and ten years later. Pre-PhD publications (and citations for these) were excluded when assessing post-PhD track records. Spearman rank correlation and Kruskal-Wallis test were computed.

**Results:**

We analyzed all together 4,790 researchers. We obtained a positive correlation between the number of Q1 publications before and after PhD (corr. coeff. = 0.21–0.54, p<0.01 in all sections), between the H-index before and after PhD (corr. coeff. = 0.32–0.56, p<0.01 in all sections), and between the citations received before and after PhD (corr. coeff. = 0.34–0.51, p<0.01 in all sections). All three metrics measured ten years after the PhD were negatively correlated with the age of the researcher at the time of obtaining the PhD (number of publications corr. coeff. = -0.09–0.22, p<0.05; H-index corr. coeff. = -0.09–0.29, p<0.08; number of citations corr. coeff. = -0.14–0.30, p<0.01). Among all disciplines, Philosophy and History and Engineering sciences show the strongest correlation between pre- and post-PhD output. When running multiple regression analysis for all three metrics as dependent variables and the number of articles, the H-index, the number of citations in the year of the PhD, the calendar year of PhD, and the gender of the researcher as independent variables, the number of articles and the H-index in the year of PhD reached the strongest positive correlations while gender had a negative correlation.

**Conclusions:**

We independently evaluated pre- and post-PhD publication performance. In connection with age, the discipline-specific reference values of scientometric parameters at the time of obtaining the PhD can help to select candidates for postdoctoral grants and positions.

## Introduction

Doctor Philosophiae (PhD) is the highest globally recognized academic qualification available in any field of research. A PhD degree can only be awarded by a university once the applicant has completed a wide-ranging and novel research project. The holder of a PhD degree can teach at universities, start independent scientific research, and also receives a higher salary as better paid positions are reserved to those with a PhD degree. For example, in Hungary only the two most basic academic (assistant lecturer) and scientific (assistant research fellow) positions can be filled by those without a PhD degree. Notably, the effect of education on wages was also sizeable in an Italian study [1], and over-education even had a negative impact [2]. The number of PhD graduates grows rapidly in all continents of the world–in some countries like China by 5% a year [3].

Commonly, the PhD student is assisted by a supervisor and the training requires 3–6 years of full-time investment. As cornerstones, completing the studying requirements, passing the doctoral examination, and the successful defense of the PhD dissertation is needed to obtain the degree. The so-called doctoral dissertation proves that the researcher can independently solve a scientific task, with which it broadens our knowledge with new, previously undiscovered results in the field [4]. The significance of the work made by PhD fellows is supported by the fact that about one third of research publications comes from a doctoral student in universities [5].

The exact conditions for obtaining the degree vary from country to country. In the UK, a PhD is awarded as part of a three-year course including specific and general subjects. Students’ dissertations are reviewed by external reviewers and an oral defense is delivered to show the candidates’ proficiency. In Australia, training with a scholarship lasts for 3–4 years [6] and publications accepted before the application can also form the basis of the doctoral thesis in certain disciplines [7]. Oral presentation for defending the dissertation is not mandatory. The United States has the longest PhD program with 4–11 years for graduates depending on subject areas. In Germany, most doctoral candidates work as employees in universities and training decisions are left to supervisors and doctoral students [8]. In Hungary, a two-year study period ending with a final exam is extended by a three-year research period, and the defense of the doctoral dissertation finishes the training.

A number of indicators are available to measure the quality and quantity of scientific output. Available bibliometric indicators include among others the total number of publications, the cumulative impact factor of all publications, the total number of citations, the number of articles with at least one citation, the number of highly cited articles, the average number of citations per article, the number of citations per year, and the H-index [9–11]. Derived bibliometric indicators have also been developed to measure researcher productivity [12]. There are no international standards for the publication requirements for obtaining a PhD [13]. The minimal requirements are mostly determined by the program, the lead tutors, and by the university. In Australia, 2–5 first or co-authored articles are required to get PhD degree [7]. There is no regulation in the United States and in Canada, although publication is strongly recommended before obtaining a PhD [13]. Peer-reviewed publication is not required in Germany. In Hungary, a discipline-specific number of publications with or without an impact factor threshold are needed to apply for a degree [14].

The scientific outcome of a doctoral training is determined by several factors in addition to the personal abilities of the doctoral student: the doctoral program, relevance, novelty, working environment, access to other experts, feasibility, and supervision [15]. The theoretical conditions of a good doctoral dissertation are also established [16]. Generally, a PhD is said to be strong in case the number of publications of the candidate is high, the total impact factor of these articles is high, and the doctoral student fulfills the training conditions in a shorter time.

To what extent does PhD training influence postdoctoral publication performance? Here, we aimed to correlate independently calculated pre- and post- PhD scientific output for a large cohort of Hungarian researchers spanning all scientific disciplines. In addition to different publication metrics we also aimed to include the age of the doctoral student to determine which features have the highest influence on a researcher’s subsequent career.

## Methods

### Database construction

Publication and citation data were downloaded from the Hungarian Scientific Bibliography (www.mtmt.hu). MTMT includes self-reported data, which is then validated at the time when one submits his or her PhD thesis. In this, we included doctors of the Hungarian Academy of Sciences (HAS), members of HAS, recipients of the Momentum grant (an ERC-grant like national scheme), researchers who have submitted Hungarian Scientific Research Fund (OTKA) applications since 2006, and researchers with university affiliation who have obtained a PhD. Unique MTMT identification numbers were used to distinguish researchers with the same name. The age of researchers as well as the age at obtaining the PhD degree was obtained from the doktori.hu public database.

### Scientific sections

HAS classifies researchers into eleven scientific sections, which are as follows: I. Language and Literature, II. Philosophy and History, III. Mathematics, IV. Agriculture, V. Medicine, VI. Engineering, VII. Chemistry, VIII. Biology, IX. Economics and Law, X. Earth sciences, and XI. Physics. Researchers can select the most relevant section based on their area of research. For researchers without a selected scientific section the designation was made based on the topics of the last five publications.

### Indicators of scientific output

We computed three indicators to measure scientific performance, the selection of these was based on our previous study [11] and availability (e.g. we had to exclude journal impact factors as these are not available for all publications). These include the *number of scientific publications*, the *H-index*, and *the number of citations* for all previous publications in the given calendar year.

*The number of all scientific publications* is based on the total number of articles published in Q1-ranked journals in the last five years as a first, last, or corresponding author. By excluding Q2, Q3, and Q4-rated articles we guaranteed that only high-quality publications are included in the database. This number reflects the contemporary scientific activity of the researcher. Q-ranking was based on the Scimago Journal Rank database (https://www.scimagojr.com/).

**Fig 1:**
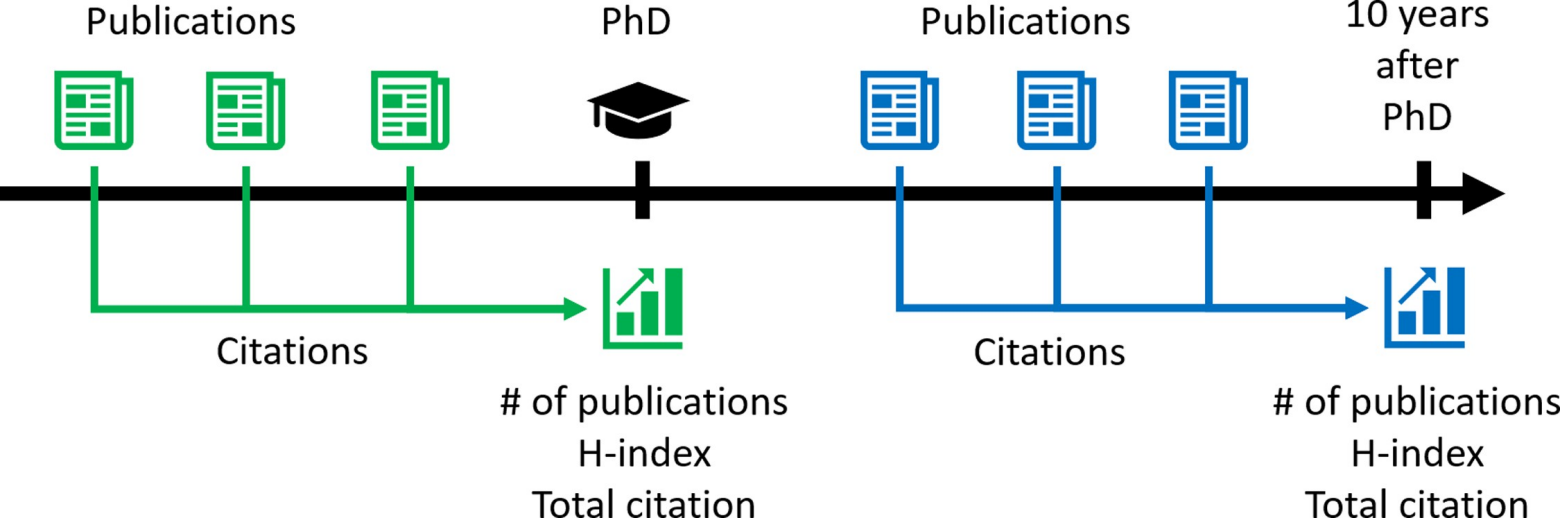
Publications before and after PhD were separately analyzed.

*The H-index* of a researcher is *n* if he/she has published *n* articles, each of which has been cited at least *n* times while there are no other articles with more than *n* citations. The number of citations includes both dependent and independent citations. This value is an indicator of an individual’s performance over his/her entire academic career and is a measure independent of the impact of journals. We have computed two different H-index values: the H-index at PhD includes all publications up to the year of the PhD award. The H-index ten years after PhD includes *only publications published after the PhD award*. The aim of this differentiation was to exclude the direct effects of publications before the PhD on subsequent H-index values (**Fig 1**).

*The total number of independent citations* to all previously accepted scientific articles in a given calendar year. Independent means that there is no overlap in the author list of the cited and the citing documents. This value is an indicator of the impact of the researcher’s former scientific activity in the present. By excluding dependent citations, it can be guaranteed that this parameter will give an objective evaluation of the researcher impact. In the same way as for the H-index, to exclude the effect of pre-PhD papers, the independent citation count was derived by using only citation received for papers published after the year of the PhD.

### Statistical analysis

The age of the researcher and all three parameters achieved in the year of the PhD (the number of articles published in the previous 5 years, H-index and number of independent citations) were compared with those values obtained ten years after the PhD. Continuous variables were compared by calculating Spearman correlation coefficients. Differences between sections were calculated using Kruskall-Wallis test. The p-value cutoff was set at p = 0.05.

## Results

### Database

**Fig 2:**
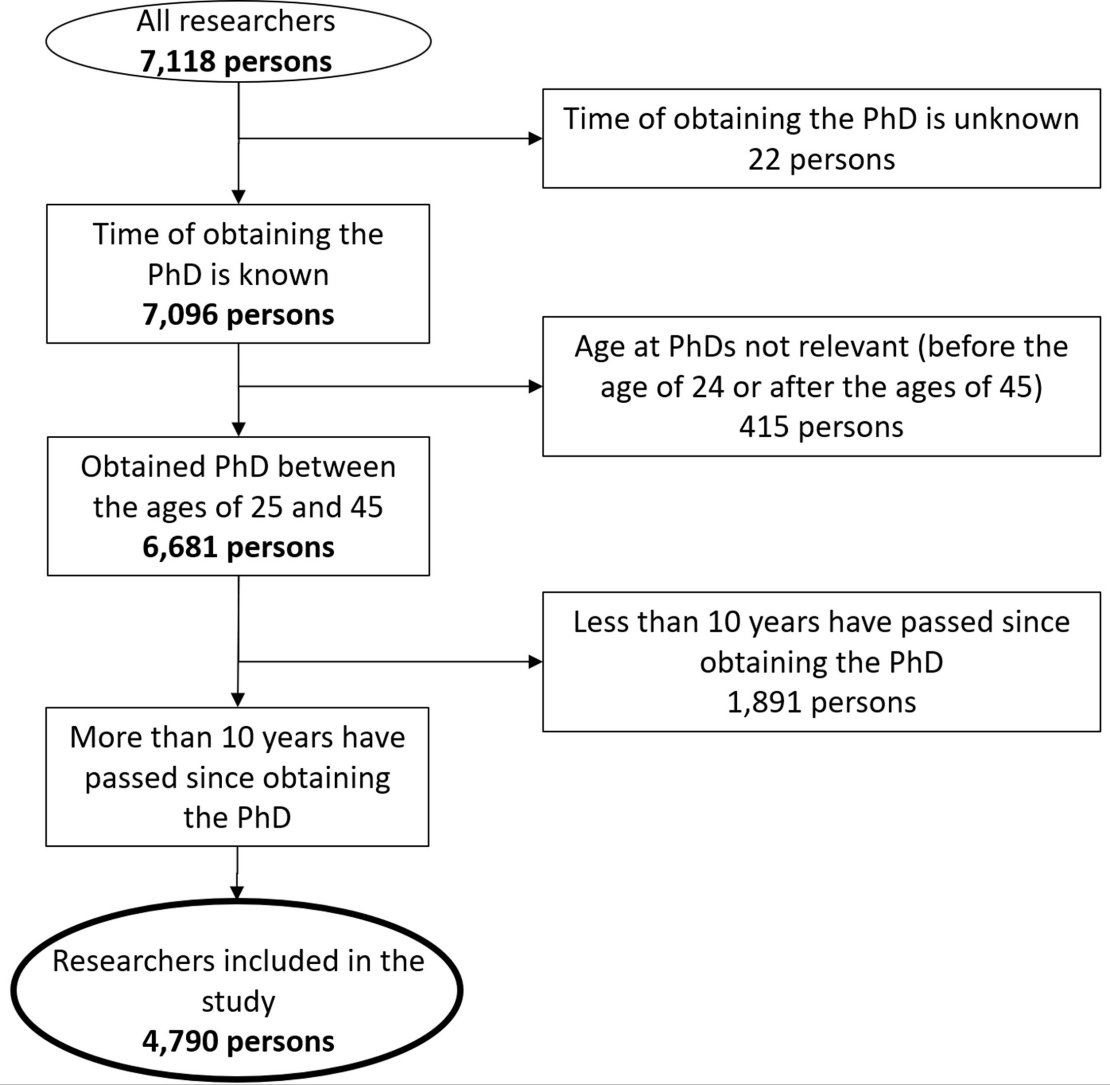
Flowchart of setting up the database up to the final cohort including 4,790 eligible researchers.

The initial database contained 7,118 researchers. Those researchers were deleted where the year of obtaining the PhD was unknown, who obtained the PhD over the age of 45 or under the age of 24. We also excluded those who obtained their degree within 10 years because we could not perform the analysis with these data. Degrees obtained over the age of 45 were excluded as these more likely refer to a former candidate degree and not to a PhD degree. Values under the age of 24 were most likely date errors in the database. The final database contains 4,790 researchers. The screening process is summarized in **Fig 2.**

The 4,790 researchers were arranged to one of eleven scientific sections of HAS, in particular, 342 researchers to Language and Literature, 457 researchers to Philosophy and History, 238 to Mathematics, 429 to Agriculture, 702 to Medicine, 360 to Engineering, 467 to Chemistry, 719 to Biology, 489 to Economics and Law, 242 to Earth sciences, and 345 to Physics. The average age of all researchers was 59.8 years, with a median of 58 years.

### Features of researchers in the year of obtaining their PhD per section

**Fig 3:**
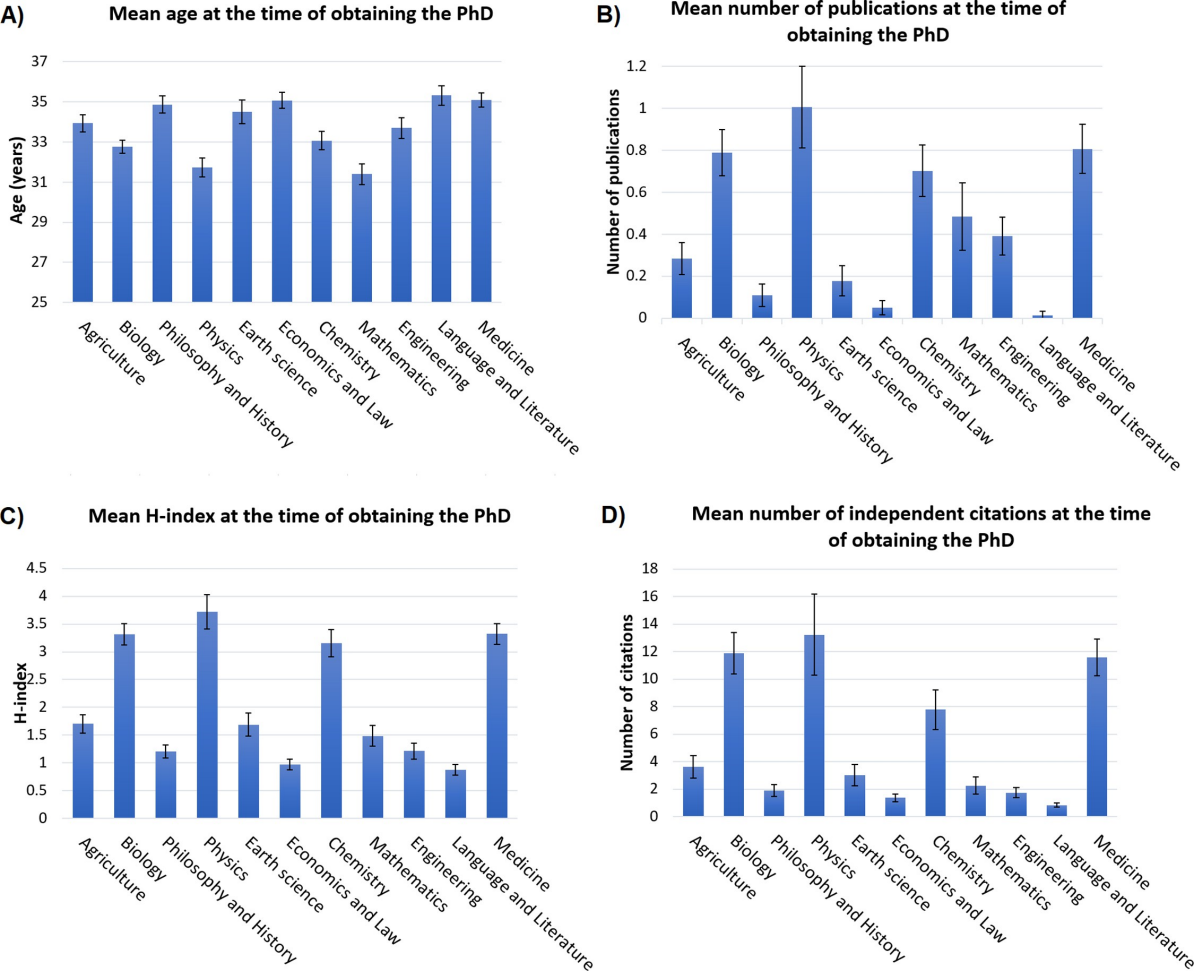
Average age at obtaining the PhD degree (A), number of publications in the previous five years (B), H-index (C), and yearly independent citations (D) at the year of obtaining the PhD in each scientific section. Mean and 95% CI are shown. See detailed data in S1 Table.

*The average age of researchers at the year of obtaining the PhD degree* was 33.8 years. There was a significant difference between scientific sections in this parameter (Kruskall-Wallis p value<1E-16). Youngest mean age at obtaining the PhD was shown in Mathematics, Physics and Biology sections (31.4, 31.7 and 32.8 years, respectively). Researchers in Language and Literature, Medicine, Economics and Law sections had the oldest average age (35.3, 35.1 and 35.1 years, respectively) (see **Fig 3A** and all the values in **S1 Table**).

There was a significant difference between sections in *the average number of peer reviewed publications in the five years preceding the PhD* (Kruskall-Wallis p value<1E-16) (**Fig 3B** and all the values in **S1 Table**). The average number of publications varied between 0.01 and 1.0. Researchers in Language and Literature, Economics and Law, and Philosophy and History sections had the lowest average values (0.01, 0.05 and 0.1, respectively). The highest average number of publications was found in Physics, Medicine and in Biology sections (1.00, 0.80 and 0.78, respectively). The average number of publications when including all researchers was 0.48.

*Mean H-index at the year of obtaining the PhD degree* in each section is presented in **Fig 3C**. Average values varied between 0.87 and 3.72. Researchers in Language and Literature, Economics and Law, Philosophy and History sections had the lowest average H-index values (0.87, 0.96 and 1.20 respectively). Highest average H-indices were found for researchers in Physics, Medicine and in Biology sections (3.72, 3.32 and 3.31, respectively). Average H-index at the year of obtaining the degree was 2.23 for all researchers.

Finally, *the number of independent citations in the year of PhD* varied between 0.83 and 13.22, and differed significantly between sections (Kruskall-Wallis p value<1E-16) (**Fig 3D** and all the values in **S1 Table**). Lowest average yearly citations were found for researchers in Language and Literature, Economics and Law and Engineering sections (0.83, 1.37 and 1.74, respectively). Researchers in Physics, Biology and Medicine sections showed the highest values for the average number of yearly citations (13.22, 11.86 and 11.56, respectively).

### Total number of articles between 5–10 years after obtaining the PhD

Correlation between *the number of articles accepted before obtaining the PhD and the number of articles accepted between 5–10 years after the PhD* was the strongest in Mathematics, Philosophy and History, and Engineering sections (Spearman corr. coeff. = 0.54, 0.48, and 0.44, respectively, p<0.01). Weakest correlation was found in Language and Literature, Medicine, and Economics and Law sections (corr. coeff. = 0.21, 0.23, and 0.26, respectively, p<0.01). Positive correlation was found between all three scientific parameters in the year of PhD and this parameter, in each section. **Table 1** shows Spearman correlation coefficients between the number of manuscripts 5–10 years after the PhD and the scientific parameters at the year of obtaining the PhD per section.

**Table 1:** Correlation between the number of publications ten years after obtaining the PhD.

	In the year of PHD:	Number of publications		H-index		# independent citations		Age	
		Corr. coeff.	p value	Corr. coeff.	p value	Corr. coeff.	p value	Corr. coeff.	p value
**Section**	Agriculture	0.30	1.68E-10	0.31	1.27E-11	0.30	1.17E-10	-0.10	0.02
	Biology	0.27	7.93E-14	0.10	0.01	0.16	5.29E-06	-0.23	3.15E-10
	Chemistry	0.27	1.08E-09	0.26	9.38E-09	0.24	1.11E-07	-0.14	1.11E-03
	Earth science	0.36	4.29E-09	0.22	2.79E-04	0.23	1.61E-04	-0.22	3.13E-04
	Economics	0.26	2.53E-09	0.13	1.95E-03	0.18	3.53E-05	-0.09	0.02
	Engineering	0.44	2.11E-18	0.41	1.39E-16	0.38	6.98E-14	-0.21	2.37E-05
	Language science	0.21	3.68E-05	0.12	0.02	0.07	0.11	-0.15	3.22E-03
	Mathematics	0.54	1.48E-19	0.22	2.37E-04	0.30	1.35E-06	-0.14	0.01
	Medicine	0.23	1.85E-10	0.23	2.17E-10	0.26	6.02E-13	-0.15	2.92E-05
	Philosophy	0.48	1.93E-27	0.29	1.20E-10	0.30	3.82E-11	-0.13	3.04E-03
	Physics	0.39	1.46E-14	0.25	1.77E-06	0.19	2.41E-04	-0.13	0.01

**Correlation between the number of publications ten years after obtaining the PhD** and scientometric parameters and age in the year of the PhD.

### H-index at ten years after obtaining the PhD

Positive correlation was found between the *H-index at the year of obtaining PhD and at ten years later* in all sections. H-index of researchers in Philosophy and History, Engineering, and Mathematics—Agriculture sections in a tie showed the strongest correlation (Spearman corr. coeff. = 0.56, 0.52, 0.51, and 0.51, respectively, p<0.01). Weakest correlation was found in Biology, Medicine and Physics sections (corr. coeff. = 0.32, 0.39 and 0.40, respectively, p<0.01). We found positive correlation between *the number of accepted manuscripts prior to PhD and value of H-index at 10 years after PhD–*except in Language and Literature section (corr. coeff. = 0.05, p = 0.18). *The number of independent citations in the year of PhD and value of H-index ten years after PhD* had strong positive correlation in all sections. **Table 2** shows Spearman correlation coefficients of the H-index values ten years after PhD and the other scientific parameters at the year of obtaining the PhD per section.

**Table 2:** Correlation between H-index ten years after obtaining the PhD degree.

	In the year of PHD:	Number of publications		H-index		# independent citations		Age	
		Corr. coeff.	p value	Corr. coeff.	p value	Corr. coeff.	p value	Corr. coeff.	p value
**Section**	Agriculture	0.34	9.78E-14	0.51	4.58E-30	0.49	8.79E-28	-0.21	5.68E-06
	Biology	0.26	1.20E-12	0.32	4.94E-19	0.35	4.47E-22	-0.24	2.96E-11
	Chemistry	0.33	5.28E-14	0.43	1.59E-22	0.44	7.53E-24	-0.15	4.23E-04
	Earth science	0.35	1.09E-08	0.48	1.85E-15	0.49	1.21E-16	-0.29	1.71E-06
	Economics	0.15	6.11E-04	0.47	4.11E-29	0.47	4.23E-29	-0.14	8.36E-04
	Engineering	0.45	5.81E-20	0.52	3.97E-27	0.49	3.02E-23	-0.27	6.95E-08
	Language science	0.05	0.18	0.45	2.30E-18	0.43	4.30E-17	-0.26	5.00E-07
	Mathematics	0.37	1.19E-09	0.51	2.31E-17	0.55	3.24E-20	-0.09	0.08
	Medicine	0.35	5.81E-22	0.39	2.39E-27	0.45	9.96E-37	-0.31	2.55E-17
	Philosophy	0.29	2.35E-10	0.56	1.18E-39	0.56	1.34E-39	-0.19	1.76E-05
	Physics	0.33	1.65E-10	0.40	3.57E-15	0.42	2.85E-16	-0.21	3.06E-05

**Correlation between H-index ten years after obtaining the PhD degree** and scientometric parameters and age in the year of the PhD.

### The number of independent citations in the tenth year after obtaining the PhD

Positive correlation was found between *the yearly number of independent citations in the year of obtaining the PhD degree and ten years later* in all sections. Researchers in Philosophy and History, Mathematics and Engineering sections showed the strongest correlation (Spearman corr. coeff. = 0.51, 0.49, and 0.47, respectively, p<0.01). Lowest correlation was found in Biology, Physics, and Language and Literature sections (corr. coeff. = 0.34, 0.36, and 0.38, respectively, p<0.01). We found positive correlation in all sections except of Language and Literature between *the number of publication prior to PhD and the yearly number of independent citations ten years after PhD*. Also, positive correlation was found between *the H-index at the year of PhD and the number of independent citations in the tenth year after PhD* in all sections. **Table 3** shows Spearman correlation coefficients for the number of independent citations in the tenth year after PhD and the other scientific parameters at the year of obtaining the PhD per section.

**Table 3:** Correlation between the number of citations in the tenth year after obtaining the PhD.

	In the year of PHD:	Number of publications		H-index		# independent citations		Age	
		Corr. coeff.	p value	Corr. coeff.	p value	Corr. coeff.	p value	Corr. coeff.	p value
**Section**	Agriculture	0.34	2.02E-13	0.45	9.07E-23	0.45	6.58E-23	-0.19	4.42E-05
	Biology	0.27	7.35E-14	0.27	4.89E-14	0.34	4.99E-21	-0.25	9.24E-12
	Chemistry	0.34	3.69E-14	0.36	2.51E-16	0.39	1.38E-18	-0.15	5.41E-04
	Earth science	0.33	4.71E-08	0.42	6.66E-12	0.45	6.76E-14	-0.30	1.04E-06
	Economics	0.17	1.00E-04	0.41	1.10E-21	0.42	1.59E-22	-0.12	4.94E-03
	Engineering	0.46	1.26E-20	0.48	1.44E-22	0.47	4.05E-21	-0.29	6.12E-09
	Language science	0.06	0.13	0.38	2.95E-13	0.38	2.22E-13	-0.24	2.55E-06
	Mathematics	0.39	2.52E-10	0.40	5.68E-11	0.49	5.34E-16	-0.14	0.01
	Medicine	0.31	1.95E-17	0.34	3.69E-21	0.42	2.51E-32	-0.27	2.30E-13
	Philosophy	0.28	3.49E-10	0.51	1.91E-31	0.51	1.76E-31	-0.18	3.99E-05
	Physics	0.34	4.79E-11	0.33	2.38E-10	0.36	1.57E-12	-0.25	1.51E-06

**Correlation between the number of citations in the tenth year after obtaining the PhD** and scientometric parameters and age in the year of the PhD

### The age of researchers at the year of obtaining the PhD and later scientific output

We found negative correlation between the age of researchers at the time of PhD and the number of publications at 5–10 years after PhD in all sections (**Table 1**). Researchers in Biology, Earth sciences and Engineering sections showed the strongest correlations (Spearman corr. coeff. = -0.23, -0.22, and -0.21, respectively, p<0.01). Weakest correlation was found in Economics and Law (corr. coeff. = -0.09, p = 0.02), Agriculture (corr. coeff. = -0.10, p = 0.02), Physics (corr. coeff. = -0.13, p = 0.01), and Philosophy and History sections (corr. coeff. = -0.13, p<0.01). We found negative correlation between *the researcher’s age at the time of obtaining the PhD and H-index ten years after the PhD* in all sections–the significance was only marginal in Mathematics (**Table 2**). Negative correlation was found between *the age of researcher at the time of obtaining the PhD and the number of independent citations in the tenth year after PhD* in all sections (**Table 3**).

### Gender specific differences

We were able to determine the gender for all researchers and compared the number of publications, the H-index, and the yearly independent citation count values reached by male (n = 3,689) and female (n = 1,101) researchers. At the time of obtaining the PhD, female students had higher mean publication count (Mann-Whitney p = 1E-07). There were no significant differences in the mean citation count and in the H-index values. Similarly, there were no significant differences between male and female researchers ten years after PhD in the three investigated scientometric parameters.

### Multiple regression

In a separate analysis we performed multiple regression by simultaneously including the number of articles, the H-index, the number of citations in the year of the PhD, as well as age, the calendar year of PhD, and the gender of the researcher for all included scientists. Dependent variables were the number of articles, the H-index, and the number of citations ten years after obtaining the PhD. In this analysis, citation in the year of PhD had no significant correlation with the number of articles (p = 0.15) and the H-index (p = 0.63). Female gender was associated with lower H-index (p<1E-50), citation count (p = 1.1E-06), and number of articles (p = 1.2E-13) ten years after PhD. The most significant positive correlations for all three dependent variables were observed for the number of articles (p = 4.4E-32 for number of articles ten years after PhD, p = 2.4E-48 for the H-index ten years after PhD, and p = 9.4E-42 for the citation count ten years after PhD) and for H-index in the year of PhD (p = 9.2E-32 for number of articles ten years after PhD, p = 1E-50 for the H-index ten years after PhD, and p = 2.6E-13 for the citation count ten years after PhD). Age and the year of PhD had minimal effects with small correlation coefficients in most settings. The detailed results for each setting including the equation values are provided in **S2 Table**.

## Discussion

Publishing during PhD training has an impact on later careers, reputation, and collaborations [17]. Here, we partially reproduced the results of Horta and Santos, but by accounting for different scientific disciplines and assessing the effects for each disciplinary area. Thus, our results not only build on and validate the previous results [17] but also significantly extend the knowledge in this field.

Early career publications are seen as a requirement to enter an academic career [18]. Along with the excellent educational and professional activity, writing strong articles is an essential condition for promotion in a scientific career [19]. However, the question remains whether scientific publication output during the PhD adequately reflects future academic performance independently of the research environment [6]? Although Horta and Santos partially responded to this question, here we performed a more in-depth analysis including different scientific disciplines. We scrutinized Hungarian researchers whether the scientific performance before the PhD influences postdoctoral scientific output. We examined three bibliometric parameters in the year of obtaining the PhD and ten years later: the number of first, last- or corresponding authored manuscripts accepted in Q1-ranked journals, the H-index, and the total number of independent citations received by the researcher in the given year. In almost all settings each of the metrics measured at the time of obtaining the PhD was positively correlated with the values measured 10 years after the PhD award. These observations are in line with our previous study analyzing publication performance of Momentum grant holders before and after grant award [9] and reinforces the validity of the Matthew effect in this setting as well.

When looking on different scientific field-specific variances, we observed the weakest correlations in Medicine, Biology, and Physics sections for all three parameters compared to other sections. Most probably, the daily routine work of physicians in patient treatment often discourages publication activity [20]. On the other hand, bibliometric indicators achieved at the year of obtaining PhD are outstanding in Medicine, as confirmed by previous other studies as well [21]. Of all scientific sections, Mathematics, Engineering, and Philosophy and History had the highest correlations between pre- and post-PhD scientific output for all three investigated indicators. These observations can be partly explained by the fact that the highest salaries for PhD holders are available in engineering, business and science fields in the United States and in Hungary which can increase compliance and engagement during PhD studies.

We obtained a strong negative correlation between age at obtaining a PhD and subsequent scientific output in all disciplines. Younger age at PhD results in significantly better postdoctoral publications with higher impact. Our results are little bit astonishing, as we have evaluated pre-PhD and postdoctoral publications completely independently. Thus, the scientometric parameters ten years after PhD only reflect the H-index, citation, and publication count of publications printed after finishing the PhD studies. Because of this separation, those who obtain a PhD at a younger age had no advantage in terms of additional years to collect citations to increase their H-index over those who acquired their PhD later.

Publication metrics are not the only indicators of the performance of an individual, but also of an institution [22], and can be used to pave the way for access to external funding sources. Therefore, a main goal of universities worldwide is to maximize research output. Different tactics can be executed to intensify publication activity: writing courses, writing support groups, and writing coaches can significantly increase the number of publications of the research participants according to a study summarizing 17 studies [23]. Reviews also help to grasp available methods for scientific writing [24]. Other options include the continuous mentoring even after the competition of the PhD [25]. Overall, the requirement to publish is a constant high pressure for employees working in academic institutions [26].

Here, we did not examine personal factors such as periods of motherhood, in which case publication activity may be paused for years. The reason for this is the lack of available data regarding maternity leaves for PhD students. Although executed in a different country, a study involving 8,544 researchers from the United States found no effect of personal circumstances including marriage, number of children, and care for aging parents on publication productivity [27]. More important factors seemed to be academic rank, salary, commitment to research, and desire for recognition, which we could not examine due to the lack of available data. The availability of funding resources can also influence publication efficiency. Reputation and experience of the supervisor [6] or of the group leader [28] also impacts the productivity of a researcher. The country-specificity of these effects should be evaluated in a future study.

We have to note a few limitations of our study. In the analysis we did not have any explanatory variable for the PhD training itself. In other words, one does not know how the PhD was trained by the supervisors, if there was co-supervision or not, if one was included in a large or small research group, if one had access to resources to do research, etc. Second, one may publish during the PhD even if the training was not focused on publication. Sometimes it all depends on the earlier training of the students, and the ability of the students. In addition, we included only Hungarian researchers. The reason for this is the utilization of the MTMT database, which only comprises data for Hungarian researchers. A main advantage of MTMT is the differentiation between dependent and independent citations. Other repositories (like Google Scholar or Scopus) either do not have this information or there is no open access. Finally, we included only Q1 articles as our focus was on research excellence and not on research quantity. One might note that lower ranked publications might still have value as scientific results. We have to note however that the majority of all publications of the investigated researchers were published in a Q1 ranked journal.

In the future, a similar study with additional data can bring more light into this field. For example, the association between time to degree, available funding, and accomplished publications during the PhD may be relevant in order to understand how funding can influence the ability to publish or not during the PhD. Conversely, funding during the PhD can affect the research performance later on.

In summary, a major novelty of our analysis is the independent analysis of pre-PhD and post-PhD scientometric parameters. Therefore, in our analysis pre-PhD publications do not directly influence the number of post-PhD citation counts and H-index. Our results are most relevant to early stage pre-PhD researchers and emphasize the importance of building a publication track record. We show that a PhD obtained at a younger age is an outstanding advantage in the later scientific career prognosticating not only more abundant publications but also higher impact of these as measured by citation counts. Post-PhD research output shows a strong correlation to the number of publications and their impact during PhD studies in all scientific disciplines. Our results emphasize the need for pre-doctoral training programs having an emphasis on regular publications. The listed scientific discipline-specific values of scientometric parameters at the time of obtaining the PhD can help to select the most suitable applicants for postdoctoral grants and positions.

## Supporting information

S1 Table Descriptive statistics for investigated parameters in each section. Mean, standard deviation, and 95% CI for the age, number of publications in the previous five years, H-index, and yearly independent citations in each scientific section at the year of obtaining the PhD (A), and ten years after obtaining the PhD (B).
(XLSX)

S2 Table Detailed results of the multiple regression analysis for the number of articles, H-index, and independent citations ten years after PhD. (XLSX)
